# Cascading predator effects in a Fijian coral reef ecosystem

**DOI:** 10.1038/s41598-017-15679-w

**Published:** 2017-11-16

**Authors:** Douglas B. Rasher, Andrew S. Hoey, Mark E. Hay

**Affiliations:** 1 Bigelow Laboratory for Ocean Sciences, 60 Bigelow Drive, East Boothbay, ME 04544 USA; 2 ARC Centre of Excellence for Coral Reef Studies, James Cook University, 1 James Cook Drive, Townsville, QLD 4811 Australia; 3 School of Biological Sciences and Aquatic Chemical Ecology Centre, Georgia Institute of Technology, 950 Atlantic Drive, Atlanta, GA 30332 USA

## Abstract

Coral reefs are among Earth’s best-studied ecosystems, yet the degree to which large predators influence the ecology of coral reefs remains an open and contentious question. Recent studies indicate the consumptive effects of large reef predators are too diffuse to elicit trophic cascades. Here, we provide evidence that such predators can produce non-consumptive (fear) effects that flow through herbivores to shape the distribution of seaweed on a coral reef. This trophic cascade emerged because reef topography, tidal oscillations, and shark hunting behaviour interact to create predictable “hot spots” of fear on the reef where herbivores withhold feeding and seaweeds gain a spatial refuge. Thus, in risky habitats, sharks can exert strong ecological impacts even though they are trophic generalists that rarely feed. These findings contextualize the debate over whether predators influence coral reef structure and function and move us to ask not if, but under what specific conditions, they generate trophic cascades.

## Introduction

Large predators generate powerful trophic cascades within many of Earth’s ecosystems^1,2^. However, the ecological roles of most large predators remain unresolved, in part because they are disappearing from nature^2–6^ but also because they are large and mobile, making them difficult to study in a rigorous manner^7^. Indeed, even charismatic predators in well-studied ecosystems, like sharks on coral reefs, remain poorly understood^8^. While they and other large predators have increasingly become the focus of ecological studies in recent time, the degree to which sharks influence the general ecology of coral reefs remains an open question^8,9^.

Many assume that reef sharks, like predators in less diverse ecosystems^10,11^, should exert strong consumptive effects that cascade to the base of the food web. Yet evidence for such effects is equivocal, even when considering reef systems where sharks remain abundant^12–16^. Equivocality may stem from differences between empirical and theoretical approaches, a focus on entire communities rather than a subset of strongly interacting species, or the fact that many seemingly pristine ecosystems are, in actuality, severely altered. Strong evidence for predator effects may also be elusive because coral reefs support complex food webs with high levels of species diversity, and potentially redundancy, within trophic levels, meaning that predator effects are dampened^17^ and unlikely to cascade under most scenarios^12^. Indeed, recent studies suggest that because reef sharks are generalist predators^18^ that hunt opportunistically within multiple reef habitats^19^, their consumptive effects are diffused across the food web and thus unlikely to cascade to impact the structure and function of the benthos^8^. However, as trophic generalists, sharks could create a strong fear of predation (non-consumptive effect) in some reef habitats^20^ such as shallow habitats where the chance of escape from a predator is reduced relative to deeper areas. Because fear effects amplify as they cascade, their ecological impacts often rival or exceed those of direct predation even where large predators consume few prey^21,22^.

Recent studies indicate that some coral reef herbivores reduce their feeding when in close proximity to reef predators or stationary predator decoys, but it is unclear if this fear causes only a temporary redistribution of herbivore feeding or an overall reduction in herbivory^23–25^. Also, some of these studies have focused on small site-attached herbivores^26^ rather than the large roving species that drive herbivory on coral reefs. As such, it remains unclear when or where large mobile predators may create fear effects that actually cascade to affect seaweed community structure and function. Theoretical and empirical studies from other ecosystems suggest that fear will cascade to shape plant communities in situations where landscape features interact with predator hunting behaviour to create areas or times of predictable risk (fear “hot spots”)^27,28^. Predictable fear hot spots diversify and structure plant communities by creating spatial escapes from herbivores where none would otherwise exist. If fear hot spots exist in some reef habitats, such as the shallow backreef, it would suggest that sharks and other large predators can play important ecological roles in these ecosystems, but that their ecological impacts are highly context-dependent. To-date, targeted investigations of this phenomenon are rare^29,30^. Here, we conducted observations and experiments in a shallow marine reserve in Fiji to explore whether large predators (>50 cm total length) create a trophic cascade through this very mechanism.

## Results

### Predation risk as a function of tide

**Figure 1:**
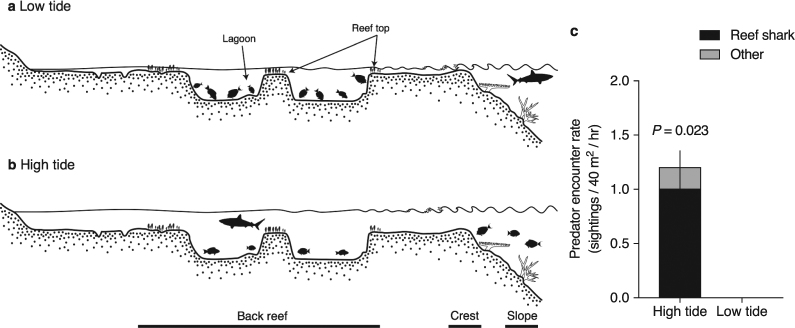
Reef topography and predation risk as a function of tide. The backreef at (**a**) low tide and (**b**) high tide. Deep, hard-bottom lagoons are habitable to large herbivorous and piscivorous (predatory) fishes throughout the tidal cycle. The upper surface of the reef (“reef top”), which has grown to mean low water mark in most places, is not accessible to these fishes at low tide. At high tide, the entire backreef becomes deeper, allowing all fishes, including large predators, access to all reef features. (**c**) Predator encounter rate (sightings/40 m^2^/hour; mean + s.e.m.) in the backreef at high vs. low tide each day (*n* = 5; paired t-test). The vast majority of predator sightings were sharks. Videos indicate that on average each 40 m^2^ section of backreef is traversed by 4–5 sharks during each diurnal high tide cycle.

High islands in the tropical Pacific are typically encircled by a fringing reef that steeply rises to a crest and then plateaus as a shallow backreef lagoon that extends from the crest to shore. Such is the case at Votua Marine Reserve in Fiji (Supplementary Fig. S1). The backreef habitat at Votua is dominated by large coral colonies (massive *Porites* spp., branching *Montipora* spp., and tabular or branching *Acropora* spp.). It is also subject to large tides (maximum amplitude ~1.8 m). Consequently, the backreef has the following topology: (a) a contiguous upper reef surface (the “reef top”) that has grown to the mean low water mark in most places and is therefore only navigable by large herbivorous and piscivorous fishes at high tide (Fig. 1; Supplementary Fig. S1), and (b) a reoccurring series of deep, hard-bottom pavements (“lagoons”) that interrupt the reef matrix; these coral-dominated lagoons remain habitable to large fishes throughout the tidal cycle (Fig. 1; Supplementary Fig. S1). During low tide, the entire backreef habitat is isolated from adjacent forereef and deep-water channel habitats. This type of habitat is common around islands throughout much of the tropical Pacific.

Using *in situ* remote video, we discovered that blacktip reef sharks (*Carcharhinus melanopterus*, ≥1.2–1.7 m TL), whitetip reef sharks (*Triaenodon obesus*, ≥1.0–1.4 m TL) and tawny nurse sharks (*Nebrius ferrugineus*, ≥1.8–2.0 m TL) make daily excursions into the backreef and do so only during high tide (Fig. 1c), presumably to capitalize on increased hunting efficiency in shallow habitats without the danger of being stranded during a low tide^31^ or to avoid encounters with larger sharks in other (deeper) habitats^32^. Large jacks (*Caranx ignobilis* and *C. melampygus*) also make forays into the backreef at high tide, but were observed infrequently (Fig. 1c). Large ambush predators such as grouper are absent from the backreef habitat. From these observations, we estimate that on average each 40 m^2^ section of backreef is traversed by 4–5 reef sharks and ~1 jack during each diurnal high tide cycle (conservatively, a 4.5 hour period).

Blacktip and whitetip reef sharks are large mesopredators that consume bony fishes (52 and 91% of diet respectively), including herbivorous fishes^18,32,33^. These sharks appear to have small home ranges^33–35^, with some individuals repeatedly visiting core areas on a daily or weekly basis^34^. They are also known from other ecosystems to predictably visit shallow lagoonal areas during high tide^34,36^. Nurse sharks consume primarily benthic invertebrates, but one-third of their diet is reef fishes^37^ and they overlap in diet niche space with blacktip and whitetip sharks^18^; thus, they too pose a predation threat to herbivorous fishes in shallow environments. Both jacks are also generalist piscivores; reef fish, including herbivores, can comprise 70–85% of their diet^38^. Therefore, tidal oscillations and predator movements interact in this backreef habitat to create a predation risk to herbivores that predictably cycles with the tide. This cycle of risk is generated almost entirely by sharks, as they accounted for ~85% of the predator sightings in our survey.

### Herbivory as a function of tide

**Figure 2:**
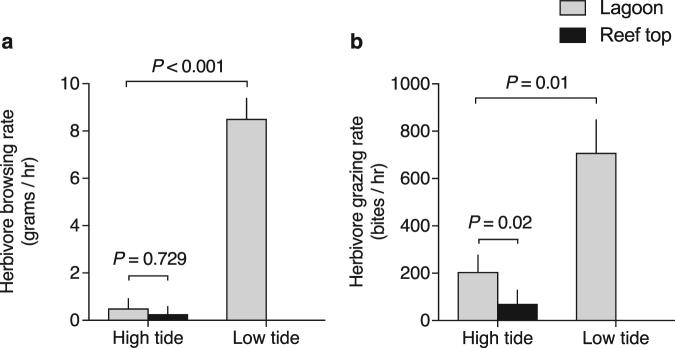
Herbivory as a function of tide. Rates of (**a**) seaweed removal by browsing fishes (grams/hour) and (**b**) substrate cropping by grazing fishes (bites/hour) in lagoons (grey bars) and on the reef top (black bars) at high vs. low tide. The reef top was not assayed at low tide, because large herbivores generally cannot access this substrate at low tide. Bars represent a mean (+s.e.m.) of five daily averages calculated for each location/tide combination. Herbivory rates in lagoons at high vs. low tide, and rates among locations at high tide, were each compared with a paired t-test.

Theory predicts that when predator encounters are variable and relatively short, prey should exhibit strong anti-predator behaviour (increased vigilance or migration) at times of high predation risk countered by energy acquisition (foraging) when safe^39,40^. Such behaviours commonly drive trophic cascades^21,27^. We therefore hypothesized that herbivorous fishes, which shape seaweed community structure on most tropical Pacific reefs^41^, should forage intensely during low tide in these habitats (when risk is low) and reduce their foraging during high tide (when risk is high). To test this hypothesis, we conducted a series of experiments where we deployed two common brown seaweeds for 2 hours surrounding the peak of each tidal phase and assessed the rate at which they were consumed by fishes. Browsing herbivores rapidly consumed seaweeds in lagoons during low tide but foraged little during high tide (Fig. 2a). When we examined the foraging rates of grazing herbivores (i.e., those species that prevent seaweed establishment by cropping or scraping the reef substratum) in hard-bottom lagoons and on reef tops, we found a similar pattern: grazing rates were significantly lower at high tide (Fig. 2b). Of the little grazing that did take place during high tide, significantly less occurred on the reef top, despite reef tops and their algal resources being wholly accessible to herbivores during this tidal period (Fig. 2b). Reef tops may be especially risky during high tide because they are structurally simple and provide few options for escape should a predator encounter occur^22,28^.

### Key herbivores and their behaviour

Almost all seaweed removal (browsing) at low tide was by the unicornfishes *Naso lituratus* and *N. unicornis* (92% and 5% of bites respectively; Supplementary Fig. S2). Most substrate grazing (94%) during both low and high tides was by the surgeonfishes *Ctenochaetus striatus* and *Acanthurus triostegus* and the parrotfishes *Chlorurus spilurus* and *Scarus rivulatus* (Supplementary Fig. S3). All are potential prey of reef sharks^18^. The marked reduction of foraging by these species during high tide (Fig. 2) may arise because they become more vigilant in the presence of sharks, or migrate to minimize shark encounters^27,28^. To investigate if these fishes migrate away from the backreef during high tide, we surveyed the herbivorous fish community across the available habitats during each tide: the entire reef at high tide, and in lagoons at low tide. Both key browsers (*N. lituratus* and *N. unicornis*) and the grazer *C. spilurus* were abundant in lagoons at low tide but were scarce (*N. lituratus* and *N. unicornis*) or half as abundant (*C. spilurus*) throughout the backreef at high tide (Table 1), indicating these species migrate, or shelter out-of-sight, presumably to minimize exposure to predators hunting in the area during high tide. Indeed, the major browsers in this ecosystem (*Naso* spp.)^42^ appear to move into the backreef on the ebbing tide and strand themselves in the lagoons to feed; they then appear to leave the backreef as the tide rises (D.B.R., personal observation). The other three key grazer species were equally abundant throughout the tidal cycle (Table 1), yet they and *C. spilurus* foraged 42–98% less during high tide (Supplementary Fig. S3), indicating key grazers withhold feeding, presumably for increased vigilance, when the chance of a predator encounter is high. Diverse anti-predator behaviours should be expected in this system given that browsing and grazing fishes differ markedly in body size, territoriality, sociality, sheltering behaviour, and home range size.

**Table 1:** Herbivorous fish biomass at high vs. low tide.

	High tide	Low tide	*P* value	Test
	Fish biomass (grams/150 m^2^)	Fish biomass (grams/150 m^2^)		
Labridae (Scarinae)	3305 ± 1614	2734 ± 283		
Acanthuridae	973 ± 143	2102 ± 666		
Siganidae	240 ± 93	173 ± 84		
Kyphosidae	71 ± 71	0 ± 0		
**Key browsers**	70 ± 40	1434 ± 801	**0.014**	Mann-Whitney
*Naso lituratus*	67 ± 37	783 ± 367	**0.014**	Mann-Whitney
*Naso unicornis*	3 ± 3	651 ± 634	0.053	Mann-Whitney
**Key grazers**	1796 ± 214	2133 ± 410	0.431	t-test
*Acanthurus triostegus*	35 ± 10	30 ± 11	0.777	t-test
*Ctenochaetus striatus*	739 ± 122	519 ± 163	0.307	t-test
*Chlorurus spilurus*	612 ± 86	1352 ± 225	**0.002**	t-test
*Scarus rivulatus*	411 ± 205	231 ± 122	0.603	Mann-Whitney

We surveyed the abundance and size of herbivorous fishes across the available habitat at high tide (the entire reef; *n* = 12) and low tide (lagoons; *n* = 6). Fish biomass (grams; calculated using known length-weight relationships) per 150 m^2^ area is reported (mean ± s.e.m.) for each herbivore family (upper section) and for species identified as the predominant browsers and grazers in the system (lower sections). Biomass at high vs. low tide was compared for each key species with a t-test or Mann-Whitney test.

### Seaweed distribution

**Figure 3:**
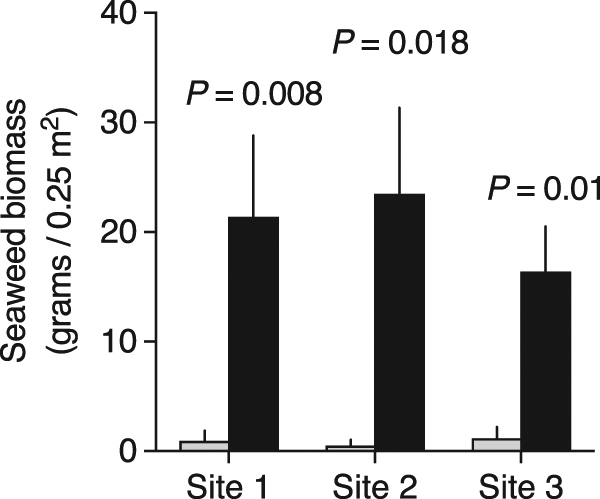
Seaweed abundance in areas of high and low predation risk. Biomass of fleshy seaweeds (grams dry mass/0.25 m^2^; mean + s.e.m.) on reef tops (black bar) vs. in adjacent hard-bottom lagoons (grey bar) in three haphazardly selected lagoonal networks. Paired censuses (<1 m apart, *n* = 10 per site) were performed on comparable substrates open to seaweed colonization. Seaweed biomass in areas of high risk for herbivores (reef tops) vs. low risk (lagoons) was compared for each site with a paired t-test or Wilcoxon test.

Demonstration of a non-consumptive trophic cascade requires evidence that not only do herbivores alter their foraging in accordance with predation risk, but also that these actions flow on to shape plant community structure. If fear effects fail to propagate downward, one would predict upright fleshy seaweeds to be generally rare on healthy coral reefs since herbivory is intense in these ecosystems. By contrast, if fear effects flow to the base of the food web, one would expect a pattern of zonation at Votua whereby seaweeds are found in fear “hot spots” that serve as spatial escapes from herbivory (i.e., the reef top) but are in low abundance elsewhere. To investigate whether seaweeds differed in abundance on reef tops vs. in lagoons (which at Votua are both hard-bottom substrates with no sand), we surveyed the seaweed community in three randomly selected lagoonal networks, comparing open substrates (i.e., not live coral cover) on the reef top to equivalent substrates in adjacent lagoons. Seaweed biomass on the reef top was ~20 times greater than in hard-bottom lagoons, where seaweeds were virtually absent (Fig. 3). Of the seaweed biomass growing on the reef top, 87 ± 4% (mean ± s.e.m.) was *Turbinaria conoides*, a brown seaweed consumed almost exclusively by *N. lituratus* and *N. unicornis* in this system^42^.

### Other factors potentially affecting seaweed zonation

Our experiments and observations suggest that shark fear effects cascade to shape seaweed distribution in this ecosystem, and do so through a few strongly interacting species in the food web. However, in the absence of a “control” site, one cannot discount the possibility that other factors influenced the seaweed zonation pattern we observed. Differences in abiotic conditions (i.e., light, physical disturbance) between reef tops and lagoons could be important. Thus, to assess the alternative hypothesis that abiotic factors created the pattern of seaweed zonation we observed, we deployed the alga most common to the reef top (*T. conoides*) onto seaweed-free, hard-bottom substrates both in lagoons and on reef tops and assessed its growth (when caged) and loss to herbivores (when not caged) over 96 hours. Growth rates of *T. conoides* did not significantly differ between lagoons (where it is generally absent) and the reef top (where it is common) (Supplementary Fig. S4). By contrast, the rate that herbivores removed *T. conoides* was more than 20 times greater in lagoons than on reef tops. Uncaged seaweeds on the reef top received negligible browsing or loss of mass owing to physical disturbance (Supplementary Fig. S4). Together, these findings indicate that variation in herbivory rather than abiotic differences likely shape seaweed distribution in this habitat. Moreover, in our previous work comparing seaweed abundance in adjacent fished and protected areas in the region, we found that brown seaweeds were abundant throughout the fished areas (i.e., were invariably abundant on hard substrates in both lagoons and on reef tops); this uniform distribution of seaweed in areas where herbivores have been overharvested^42^ lends credence to our interpretation that abiotic differences across the reef did not create the patterns of seaweed zonation we observed in Votua Marine Reserve.

It is also important to consider that reef tops are exposed to herbivores for less time than are lagoons each day. If reef herbivory rates were similar throughout the tidal cycle, this shorter exposure could enhance seaweed accumulation on the reef top. However, even when the reef top is accessible to herbivores (at high tide), this seaweed-rich area receives little grazing or browsing (Fig. 2) owing to shifts in herbivore behaviour (Table 1, Supplementary Fig. S3). Further, herbivory rates in lagoons are very low at high tide as well (Fig. 2). As such, all lines of evidence indicate that tidal oscillations, reefscape topography, and herbivore fear of predators *interactively* shape seaweed distribution in this system. It is this interaction that makes the observed trophic cascade habitat- and context-dependent.

## Discussion

There is little evidence that large predators consistently produce trophic cascades on coral reefs. However, past studies of trophic cascades in these ecosystems have largely focused on the *consumptive* effects of predators (which are diffuse) and have generally done so using coarse community assemblage data. Thus, it remains unclear how much large predators may impact reef structure or function through direct versus indirect (fear) effects or how this may vary with location and habitat type. It is possible that predator effects can cascade, but happen through non-consumptive instead of consumptive mechanisms, and do so only in particular habitats and among a subset of strongly interacting species.

By resolving reefscape effects and focusing on strongly interacting species^43^, we found phenomenological evidence that sharks, via the fear they exert, can indirectly shape seaweed distribution in a shallow backreef habitat. Our findings extend those generated from studies employing predator decoys and statistical models^23–26^ to indicate that periodic visits by large predators can have *lasting* impacts on reef seaweed community structure, but only under certain contexts^8^. We show that where reefscape topography and tidal oscillations channel predator movements to predictable times and places (i.e., create fear “hot spots”), trophic cascades can arise. Such findings give context to the debate over whether trophic cascades can exist on coral reefs and move us to ask not if, but under what particular conditions, they might occur.

When and where trophic cascades arise will depend on how reefscape configuration, predator diversity, predator hunting mode, and prey behaviour interact to shape the rules of engagement between predators and prey^27,28^. For instance, trophic cascades are not expected on most deep forereefs, where predators abound and shelter availability and foraging risk do not vary with the tides. In such places reef shark encounters are less predictable and herbivore vigilance is eventually overridden by the need to acquire resources^39,40^. Indeed, forereefs where mesopredators abound are not overgrown by fleshy seaweeds^16,44^. By contrast, trophic cascades are more likely to exist in shallow backreef habitats or among lagoonal patch reefs where reefscape features and abundant predator populations create places and/or times of known risk to herbivores. Given the backreef we studied is typical of fringing reefs that surround high islands throughout the central and western tropical Pacific, we expect these trophic cascades to exist wherever similar biological and physical conditions occur throughout the region^45^.

Cascades of fear are rarely considered in resource management^22^ but this and other reef studies^30^ provide reason to do so. At Votua, large predator movements appear to shift the timing with which herbivores visit and feed in the backreef, such that some herbivores of high food value (e.g., *Naso unicornis*) are concentrated in lagoons at low tide. Efficient human predators^46^ can easily harvest these large herbivores at low tide, since they have limited ability to escape. Indeed, outside of Votua Marine Reserve, human predators have dramatically reduced the abundance of all large herbivores in the backreef, triggering a phase shift to seaweed dominance within the fishing areas^42^. With knowledge of predator movements and resultant herbivore migrations, resource managers could mitigate this negative human impact in similar ecosystems by regulating not only where but *when* herbivores are harvested. Our example highlights the need to consider predator effects in ecosystem-based management^20,47^.

The trophic cascade we describe here is similar to those in seagrass^48^ and montane forest^49–51^ ecosystems in that each involve large vertebrate carnivores and herbivores, are mediated in part by herbivore behaviour, and manifest through interactions with the landscape. While this sample size is small, the fact that such parallels exist among markedly different ecosystems raises the intriguing question of whether predators created important “landscapes of fear” in many of Earth’s ecosystems prior to the Anthropocene^7^. Unfortunately, such a question is difficult to answer because studies of trophic cascades involving large vertebrate predators have rarely been performed^52,53^, and humans have functionally eliminated large carnivores from many of Earth’s ecosystems^2–7^. For example, large apex predator sharks^8,9,54^ as well as the large mesopredator reef sharks studied here^55^ are now generally rare or absent on coral reefs exposed to heavy fishing pressure; thus, the effects we documented may already be extinguished from many places. Despite these difficulties we need to study Earth’s remaining wild places where predators still abound, and capitalize on chance events and variability in nature, if we are to reveal the ecological roles of predators^2^. Only then can we understand the ramifications of predator loss or recovery.

## Methods

### Experimental design

We conducted our study in July and August of 2012 on a shallow, coral-dominated, lagoonal backreef adjacent to Votua Village, Korolevu-i-wai, Viti Levu, Fiji (18°13.049′S, 177°42.968′E). Typical of the fringing reefs that surround high islands in the tropical Pacific, the forereef at Votua grades steeply to a reef crest, then forms a shallow backreef lagoon that extends to shore (Fig. 1; Supplementary Fig. S1). The biogenic habitat in the backreef, which is comprised primarily of massive *Porites* spp., branching *Montipora* spp., and tabular or branching *Acropora* spp. corals, has grown vertically to meet the low tide limit in many places; however, the reef is interrupted regularly by deep, hard-bottom pavement areas (“lagoons”) that are populated by corals and that remain submerged throughout the tidal cycle (~1 + m deep at low tide, ~2–2.8+ m deep at high tide; Fig. 1, Supplementary Fig. S1). The upper surface of the reef (“reef top”) is comprised of a relatively even mixture of living coral and dead coral substratum covered by crustose coralline algae, small algal turfs, and large fleshy macroalgae (hereafter “seaweeds”). As a consequence of a large tidal range (~1.0–1.8 m), the reef top is mostly inaccessible to large herbivorous and piscivorous fishes at low tide (Fig. 1a,b). Lagoons remain habitable by large fauna throughout the tidal cycle, however the backreef is completely isolated from the greater reefscape (i.e., the forereef and deep-water channels) at low tide (Fig. 1a; Supplementary Fig. S1). The duration of both tidal phases is similar each day.

In 2002, Votua Village established a no-take marine reserve extending from shore to the forereef. Overfished and seaweed-dominated at the time of establishment, the backreef within the reserve has since recovered, as has its associated fish stocks, including large herbivorous fishes that shape the abundance and distribution of seaweed in this system^42,56^. Consequently, rates of browsing (seaweed removal) and grazing (substrate cropping) by herbivorous fishes are high within the reserve and seaweeds are relatively scarce^42^. Reef sharks are also common to the reserve, in that they make daily forays into the backreef at high tide (see Results, Fig. 1c), presumably to hunt^31^ prey including herbivorous fishes^18,37^. Large piscivorous jacks (family Carangidae)^38^ are also seen in the backreef at high tide, though much less frequently (see Results, Fig. 1c). These predators are not found in the reserve at low tide. Large ambush predators such as grouper have been absent from the backreef for the 7+ years in which we’ve studied these reefs (D.B.R., personal observation). Therefore, under these physical and biological conditions, risk of predation to herbivores (1) varies with tide, such that risk is low at low tide and high at high tide, and (2) is driven almost entirely by potential shark encounters (see Results, Fig. 1c). It is in this context that we made observations and conducted experiments to investigate the possibility of a trophic cascade.

### Quantifying predator activity as a function of tide

To quantify the rate that large predators known to consume herbivorous fishes (sharks, jacks, snapper, grouper, barracuda; >50 cm total length) are encountered in the backreef as a function of tide, we deployed video cameras (Hero 1 and 2, GoPro Inc.) for a 2-hour period surrounding the peak of each diurnal high and low tide every day (see details below, *n* = 4 cameras per low tide per day; *n* = 8 cameras per high tide per day) for six days – three in which high tide occurred in the morning and 3 in which it occurred in the afternoon - and scored the number of predator sightings in each video. We excluded videos from 1 August due to storm swell that interfered with camera deployment (thus *n* = 5 days). We screened each 2-hour video at 4x playback speed. Human activity was present in the field of view during the deployment of the assay (i.e., roughly the first one to four minutes of footage); we omitted this footage and waited for an additional ~30–60 seconds, after fishes in the field of view resumed their normal swimming and feeding behaviour, before we commenced scoring the video. We also excluded the footage (and the ~60 seconds before it) where humans entered the field of view to retrieve the assay. Humans were not otherwise present in the area during the assay. When a relevant predator was spotted in the field of view (reef top deployments: ~4 m^2^; lagoon deployments: ~6 m^2^), we commenced normal playback speed and recorded the identity and size of the predator and the duration of its visit. As a conservative measure, in instances where it appeared that the same individual predator may have visited a single camera repeatedly during an assay, or visited several cameras in rapid succession, we scored the predator sighting once. Omitting this conservative measure would only strengthen the pattern we documented, as predators were observed exclusively at high tide (Fig. 1).

Large predator sightings consisted primarily of sharks (the reef sharks *Carcharhinus melanopterus* and *Triaenodon obesus* and the tawny nurse shark *Nebrius ferrugineus*) and, in a few instances, jacks (*Caranx ignobilis* and *C. melampygus*). Some may consider excluding *N. ferrugineus* from our survey because its diet consists primarily of invertebrates^37^. However, a large proportion (≥30%) of its diet is also reef fishes, it feeds at a high trophic level, and its niche overlaps with other piscivorous reef sharks^18,37^; hence, *N. ferrugineus* poses a potential threat to herbivorous fishes when encountered. For this reason, we retained *N. ferrugineus* sightings in our analysis. Even if we excluded nurse sharks from our analysis, the pattern we report (Fig. 1) remains significant. We scaled the total number of predator sightings observed in each video by time (video duration). For a given tide and day, these rates (sightings per hour) were then summed across cameras and scaled by sampling effort (total area of reef monitored) to calculate a total predator encounter rate for each tide (since herbivores may integrate risk across all potential encounters). Encounter rates (sightings per 40 m^2^ per hour) at high vs. low tide each day (*n* = 5) were compared with a paired t-test.

### Quantifying herbivory as a function of tide

Herbivorous fishes strongly influence algal abundance and distribution on most tropical Pacific reefs^41^. Although diurnal patterns of fish herbivory in backreef habitats are well established^57^ (i.e., increasing in the morning, peaking mid-day, declining in the afternoon, and diminishing at dusk), it is unclear within this context whether herbivory rates vary with tidal phase. To quantify whether these herbivores change their rates of foraging throughout the tidal cycle (i.e., at times of high vs. low predation risk), we conducted a series of feeding experiments where we documented rates of herbivory (browsing and grazing) on the reef as a function of tide (diurnal high tide vs. low tide) and space (reef top vs. lagoon). To avoid confounding tide with time of day, we replicated the experiment six times over two separate 3-day periods, one in which high tide occurred in the morning and the other in which high tide occurred in the afternoon. As with our predator surveys, we excluded all data from August 1 due to a storm that interfered with our ability to collect reliable data.

Each day we deployed two common brown seaweeds (*Sargassum polycystum* and *Hormophysa cuneiformis*) (a) in lagoons and on the reef top within 2 hours of peak high tide (*n* = 4 per location) and (b) in lagoons within 2 hours of peak low tide (*n* = 4) and measured the total amount of seaweed consumed by herbivores relative to caged seaweeds (controls) deployed in the same area. Seaweeds were not deployed on the reef top at low tide because this habitat is inaccessible to large herbivores at low tide. We used the seaweeds *S. polycystum* and *H. cuneiformis* in the assay because they are targeted by the same herbivores as *Turbinaria conoides*, the dominant alga on the reef tops in the reserve, but are consumed more rapidly to the extent that they are rare in the reserve and very common in the adjacent fished areas. This allowed us to make rapid assessments of browsing among the principal consumers of *T. conoides* without our assessments being confounded by background levels of the same seaweed in the environment. Seaweeds were spun 20 revolutions in a salad spinner and weighed before each experiment (mean treatment wet mass ± s.e.m.; *H. cuneiformis* = 25.87 ± 0.44 g; *S. polycystum* = 26.62 ± 0.55 g). Replicates consisted of a single thallus of each species, the holdfasts of which were woven ~15–20 cm apart into a 50 cm section of three-stranded nylon rope attached flush to the substrate. Replicates were deployed ≥10 m apart and dispersed among several lagoonal networks. Reef top deployments were positioned at least 0.5 m away from any lagoon edge and in areas devoid of seaweed so as to make them as apparent to herbivores as the seaweeds deployed in lagoons. A small cage containing the same amount of each seaweed species (weighed and roped as above) was also paired with each treatment replicate (≤3 m away) to control for any change in the mass of treatment seaweeds due to factors other than herbivory. Experiments were conducted during calm seas.

After the experiment, replicates were bagged individually and returned to the lab where they were spun and re-weighed. The biomass of *S. polycystum* and *H. cuneiformis* consumed by herbivores in each treatment/control pair were each calculated using the equation [T_i_ × (C_f_/C_i_)] − T_f_, where T_i_ and T_f_ are the initial and final masses (respectively) of a seaweed exposed to herbivory and C_*i*_ and C_*f*_ are the initial and final masses (respectively) of its paired caged control. The total mass (*S. polycystum* and *H. cuneiformis* combined) of each replicate consumed per hour was calculated; rates were then averaged among replicates from each location and tide within a day to produce a daily average browsing rate for each location and tide (*n* = 5). To assess whether browsing in lagoons varied with tide, we compared the rate that herbivores consumed seaweed in lagoons at high vs. low tide each day with a paired t-test. To assess whether browsing in lagoons at high tide was less (see Results, Fig. 2) simply because it was redistributed to the reef top, we compared the rate that herbivores consumed seaweed in lagoons vs. on the reef top at high tide each day using a paired t-test.

To assess rates of grazing on the substratum (a process that prevents seaweed establishment), we deployed GoPro video cameras in front of reef substrate harbouring the epilithic algal matrix (“EAM”; an assemblage of filamentous algal turfs, crustose algae, seaweed germlings, cyanobacteria, microbes, and detritus growing on the reef substratum) during each 2-hour feeding experiment and recorded herbivore bites on the EAM (*n* = 3 cameras per location per tide per day) within a 1 m^2^ area in front of each camera. We haphazardly selected a starting point in each video and scored 1 hour of footage, documenting the identity and size (to nearest 5 cm increment) of each nominally herbivorous fish that entered the 1 m^2^ area and the number of bites it took from the EAM. We excluded any footage interrupted by human activity as described above. While small size-class fish (juveniles) are a common component of reef shark diets^18^ and thus may be subject to fear effects [but see ref.^25^], their contribution to the process of herbivory on coral reefs is relatively minor^58^. Moreover, they are difficult to identify to species from video footage. Hence, individuals <10 cm in total length were not counted. The total number of bites by all herbivores in each 1-hour video was calculated; totals were then averaged among replicates from each location and tide within a day to produce a daily average grazing rate for each location and tide (*n* = 5). For the four herbivores found to be the predominant grazers during both tidal phases (the surgeonfishes *Acanthurus triostegus* and *Ctenochaetus striatus* and the parrotfishes *Chlorurus spilurus* and *Scarus rivulatus*; totalling 94% of all bites), we calculated species-specific grazing rates using the same approach. Community-level rates of grazing in lagoons at high vs. low tide each day were compared with a paired t-test. Likewise, rates of grazing in lagoons vs. on the reef top at high tide each day were compared with a paired t-test.

### Identifying key browsers and their rates of seaweed consumption

In addition to estimating the rate with which herbivores removed seaweed biomass during each tide (see section above), we also deployed GoPro video cameras in front of every browsing assay to document the identity of the herbivores responsible for seaweed removal and their relative importance to the process (*n* = 4 cameras per location per tide per day). Of the cameras deployed at each location during each tide, we randomly selected and scored one replicate video. Thus, we scored five browsing videos per location and tide (1 per location per tide per day × 5 days = 5). For each video, in the first 60 minutes of footage absent human activity (see above for method of discounting footage of assay deployments and retrievals) we documented the identity and size (to nearest 5 cm increment) of each nominally herbivorous fish that fed from either *S. polycystum* or *H. cuneiformis* and the number of bites it took from each alga (total bites = 4,690). We did not score fishes <10 cm total length for the reasons described above. We tabulated the total number of bites (*S. polycystum* and *H. cuneiformis* combined) taken by each fish species in each video so as to calculate species-specific browsing rates (bites per hour) for each location and tide (*n* = 5). However, we limited our comparison of browsing rates to those calculated for fishes in lagoons at low tide since 99% of browsing occurred during low tide.

### Quantifying herbivore community structure throughout the tidal cycle

To determine if herbivorous fishes alter their distribution depending on tidal state, we surveyed the abundance and size of herbivorous fishes across the available habitats at high and low tides: the entire reef at high tide and lagoons at low tide. The two surveys were both conducted within 2 hours of peak tide and at approximately the same time of day. To achieve similar timing, the survey of the backreef at high tide was conducted three days prior to the feeding experiments (see above) and a week prior to the lagoon survey. The survey of the backreef at high tide consisted of three “rows” (outer, middle, and inner reef) of four 30 × 5 m transects (*n* = 12 transects total); each row was set parallel to shore and separated by 10–20 m. The survey of lagoons at low tide consisted of six 30 × 5 m transects run along the middle contour of several large lagoonal networks, with the start and end of each transect spaced by ≥10 m. For each transect, a single snorkeler (A.S.H.) slowly swam while simultaneously deploying the transect tape and identified all nominally herbivorous fishes (families: Labridae (subfamily Scarinae), Acanthuridae, Siganidae, Kyphosidae) within the 5 m band and recorded their body length (to the nearest 5 cm size class category). The biomass of each individual was estimated from its length, using defined length-weight relationships; species biomass totals were summed for each transect. For species identified as key browsers and grazers in the system (see Results, Supplementary Fig. S2 and S3), their biomass (grams per 150 m^2^ of reef area) at high vs. low tide was compared with a t-test or, where data were not normal but met the other assumptions of the t-test, a Mann-Whitney test^59^.

### Quantifying patterns of seaweed distribution and the factors controlling distribution

We surveyed the abundance and distribution of seaweeds in the backreef of Votua, focusing on areas of high predation risk (the reef top) vs. relative safety (lagoons). To do so, we scored the identity and biomass of seaweeds growing on the reef top and in adjacent lagoons in three sets of lagoonal networks. Lagoonal networks were haphazardly selected on Google Earth. These were separated by >200 m, not connected, and thus served as independent tests. Within the centre of each, we selected a random compass bearing and swam that bearing until we encountered the lagoon edge. At the edge, we deployed a 50 × 50 cm quadrat on the nearest reef top that was available for seaweed colonization (i.e., not live hard coral, soft coral, or sponge). All upright seaweeds within the quadrat were harvested and bagged. We then deployed the quadrat in the nearest topographic low point of the adjacent lagoon (<1.5 m away) that was reef substrate available for seaweed colonization (i.e., not sand, rubble, or the organisms listed above) and harvested seaweed from the quadrat. Ten paired surveys were conducted in each lagoonal network. Samples were immediately returned to the laboratory where they were sorted, identified to genus, and dried at 65 degrees Celsius to a constant mass. The total dry mass (grams ± 0.01) of seaweed on the reef top vs. in lagoons was compared for each site using a paired t-test, except in one instance (Site 1) where the data were not normal but did meet the other assumptions of the test; these were instead evaluated with a Wilcoxon test^59^.

To investigate whether the seaweed zonation pattern we documented (Fig. 3) was due to herbivory rather than abiotic factors, we conducted an additional experiment using the seaweed *T. conoides*, the most abundant alga on upper reef surfaces (87 ± 4% of seaweed dry mass on the reef top, mean ± s.e.m.). In this experiment, we deployed replicate assays as pairs–one deployed on the reef top and the other <3 m away in an adjacent lagoon (*n* = 10 pairs)–and assessed the amount of *T. conoides* consumed by herbivores after 96 hours. Although seaweeds are commonly found on reef tops, we specifically selected areas on the reef top where seaweeds were rare or absent to reduce the potential for surrounding seaweeds to mask the presence of the experimental assay. Treatment replicates consisted of three seaweed thalli that were spun, weighed (mean wet mass ± s.e.m.: 27.12 ± 0.49 g), and woven 15 cm apart into a 50 cm section of three-stranded nylon rope. In addition, identical replicates (mean wet mass ± s.e.m.: 27.61 ± 0.49 g) were placed in cages and deployed within 1 m of each assay to (a) assess differences in *T. conoides* growth among locations and (b) account for mass changes in treatment seaweeds due to factors other than herbivory (i.e., to serve as a control). Blocks of replicates were spaced 8–10 m apart, spanning a large interconnected network of lagoons. We calculated the amount of seaweed consumed by herbivores using the equation above. The rate that herbivores consumed *T. conoides* in lagoons vs. on the reef top, and the rate that caged *T. conoides* grew in lagoons vs. on the reef top, were each compared with a paired t-test.

### Statistical analysis

Statistical analyses are described in detail within each section. Unless noted, data conformed to test assumptions. All tests were performed in SigmaStat v. 3.5 (Systat Inc.).

### Data availability

Data reported in this paper are archived at Dryad (http://datadryad.org).

### Research ethics

All methods were carried out in accordance with relevant guidelines and regulations. Research was conducted under a research permit issued by the Fijian Government.

## Electronic supplementary material

Supplementary Information
