# Supplementary material for: Cascading predator effects in a Fijian coral reef ecosystem
Source: Sci Rep. 2017 Nov 16;7:15684. doi: 10.1038/s41598-017-15679-w (PMC5691076; doi:10.1038/s41598-017-15679-w)
Supplement: Supplementary file 1 — Supplementary Information [file 41598_2017_15679_MOESM1_ESM.docx]

**Supplementary information**

**Title**

Cascading predator effects in a Fijian coral reef ecosystem

**Authors**

Douglas B. Rasher^1*^, Andrew S. Hoey^2^, Mark E. Hay^3^

**Author affiliations**

^1^Bigelow Laboratory for Ocean Sciences, 60 Bigelow Drive, East Boothbay, ME, 04544, USA

^2^ARC Centre of Excellence for Coral Reef Studies, James Cook University, 1 James Cook Drive, Townsville, QLD, 4811, Australia

^3^School of Biological Sciences and Aquatic Chemical Ecology Centre, Georgia Institute of Technology, 950 Atlantic Drive, Atlanta, GA, 30332, USA

**Supplementary Figure S1 | Topography of the backreef at Votua.** (**a**) Aerial view of Votua Marine Reserve, which encompasses the shallow, coral-dominated backreef that extends from the reef crest to the shore. The backreef habitat is comprised of massive coral features (dark shaded areas), the majority of which have grown to mean low water mark, and relatively deep, hard-bottom lagoons (light areas), which remain submerged throughout the tidal cycle. (**b**) A close-up picture of a deep lagoon, captured from video footage of a feeding assay conducted around low tide. Note the reef top in the background and its reflection on the water’s surface.

Photo credits: (**a**) Google Earth, Image (c) 2017 CNES/Airbus; (**b**) D.B. Rasher

**Supplementary Figure S2 | Agents of seaweed removal at low tide.** Browsing rates (bites / hour; mean + s.e.m.) at low tide were estimated for each herbivore species using video cameras deployed in front of two common brown seaweeds, *Sargassum polycystum* and *Hormophysa cuneiformis* (*n* = 5)*.* Ninety-nine per cent of all browsing occurred around low tide.

**Supplementary Figure S3 | Grazing rates of key herbivores at high vs. low tide.** Rates of substrate grazing (bites / hour) in lagoons (grey bars) and on the reef top (black bars) at high vs. low tide by the surgeonfishes (**a**) *Acanthurus triostegus* and (**b**) *Ctenochaetus striatus* and the parrotfishes (**c**) *Chlorurus spilurus* and (**d**) *Scarus rivulatus*. No reef top evaluations were made during low tide, as large herbivorous fishes generally cannot access the reef top at low tide. Bars represent the mean (+ s.e.m.) of five daily averages calculated for each location/tide combination. Fish species **a**-**d** accounted for 94% of all grazing recorded.

**Supplementary Figure S4 | Additional test of the factors controlling seaweed zonation.** The rate (grams / 96 hours; mean + s.e.m.) with which the brown alga *Turbinaria conoides* (**a**) grew inside cages and (**b**) was consumed by herbivores outside of cages, when deployed in lagoons vs. on the adjacent reef top for 96 hours (*n* = 10). Initial masses (mean ± s.e.m.) of caged and uncaged seaweeds were 27.61 ± 0.49 and 27.12 ± 0.49 grams, respectively. The rate with which *T. conoides* grew in lagoons vs. on the reef top, and the rate with which herbivores consumed the alga in these locations, was each compared with a paired t-test.
